# *Mycobacterium tuberculosis*-infected human monocytes down-regulate microglial MMP-2 secretion in CNS tuberculosis via TNFα, NFκB, p38 and caspase 8 dependent pathways

**DOI:** 10.1186/1742-2094-8-46

**Published:** 2011-05-11

**Authors:** Justin A Green, Shruti Dholakia, Karolina Janczar, Catherine WM Ong, Rachel Moores, Julie Fry, Paul T Elkington, Federico Roncaroli, Jon S Friedland

**Affiliations:** 1 Section of Infectious Diseases and Immunity and the Imperial College Wellcome Trust Centre for Clinical Tropical Medicine, Hammersmith Campus, Imperial College London, London, W12 0NN, UK; 2 Section of Neuropathology, Department of Medicine, Imperial College London, London, W12 0NN, UK

## Abstract

Tuberculosis (TB) of the central nervous system (CNS) is a deadly disease characterized by extensive tissue destruction, driven by molecules such as Matrix Metalloproteinase-2 (MMP-2) which targets CNS-specific substrates. In a simplified cellular model of CNS TB, we demonstrated that conditioned medium from *Mycobacterium tuberculosis*-infected primary human monocytes (CoMTb), but not direct infection, unexpectedly down-regulates constitutive microglial MMP-2 gene expression and secretion by 72.8% at 24 hours, sustained up to 96 hours (P < 0.01), dependent upon TNF-α. In human CNS TB brain biopsies but not controls the p38 pathway was activated in microglia/macrophages. Inhibition of the p38 MAP kinase pathway resulted in a 228% increase in MMP-2 secretion (P < 0.01). In contrast ERK MAP kinase inhibition further decreased MMP-2 secretion by 76.6% (P < 0.05). Inhibition of the NFκB pathway resulted in 301% higher MMP-2 secretion than CoMTb alone (P < 0.01). Caspase 8 restored MMP-2 secretion to basal levels. However, this caspase-dependent regulation of MMP-2 was independent of p38 and NFκB pathways; p38 phosphorylation was increased and p50/p65 NFκB nuclear trafficking unaffected by caspase 8 inhibition. In summary, suppression of microglial MMP-2 secretion by *M.tb*-infected monocyte-dependent networks paradoxically involves the pro-inflammatory mediators TNF-α, p38 MAP kinase and NFκB in addition to a novel caspase 8-dependent pathway.

## Background

Central nervous system (CNS) tuberculosis (TB) is heavily over represented in mortality figures causing over 30% of adult TB deaths [1,2]. This is because the marked CNS inflammatory response to the pathogen is poorly tolerated. CNS TB is an encephalomyelitis with invasion of parenchymal brain tissue. The mechanisms resulting in CNS invasion and tissue destruction are poorly defined but are known to involve pathogen-driven host-derived factors [3,4].

Matrix metalloproteinases (MMPs) are a family of 23 zinc containing endopeptidases that degrade extracellular matrix, facilitate leukocyte recruitment, process cytokines and chemokines, as well as cleave cell surface molecules leading to intracellular signaling events [5]. MMP activity is controlled at the transcriptional level and by tissue inhibitors of metalloproteinase (TIMP-1 to -4), as well as by compartmentalization and secretion of pro-forms (zymogens). MMPs have a pivotal role in diseases with marked inflammatory phenotypes such as rheumatoid arthritis [6], sarcoidosis [7] and atherosclerosis [8] as well as CNS disorders like multiple sclerosis [9] and HIV encephalitis [10]. There is growing evidence on their role in the pathogenesis of TB [11-14]. An emerging concept is that important balances exist, not only between MMPs and TIMPs, but also between different MMPs with similar substrate affinity meaning that changes in MMP concentrations may be an important regulatory mechanism [15,16].

Control of gene regulation by binding of transcription factors such as NFκB is key in control of specific MMPs. Most MMPs (e.g. MMP-1, -3, -7, -8, -9, -11, -12, -13, -19 and -26) have a TATA box at -30 bp from the transcriptional start site. However, MMP-2, -14 and -28 lack the TATA box often resulting in high levels of constitutive expression [17-19]. Steroids, which are transcriptional regulators of MMP gene expression, have a clinically proven beneficial effect in CNS TB which we have shown may be due in part to their influence on MMP secretion [20,21]. Additionally, upstream signaling molecules including the p38 mitogen activated protein kinases (MAPK) regulate both MMP gene transcription and post-transcriptional gene stability [22,23]. Proteolytic cleavage of these kinases and signal transducers by caspases is usually an inactivation step during apoptosis. However, some kinases such as MEK kinase-1 are activated by caspase-3 mediated cleavage [24]. There are few published data on the control of MMP secretion by caspases although caspase 8 has been reported to phosphorylate STAT1 and thus regulate IFN-γ suppression of MMP-9 secretion [25]. Additionally caspases may induce apoptosis via an MMP-3 dependent process [26].

MMP-2 degrades basement membrane, type IV collagen, gelatin, aggrecan and laminin [27]. Nonsense mutations in the human MMP-2 gene result in phenotypic changes defined clinically as the Torg, Winchester and Nodulosis-Arthropathy-Osteolysis syndromes [28]. MMP-2 is constitutively expressed at high levels by many cells and is regulated by pro-peptide activation [12,18]. In the CNS, MMP-2 drives neuronal apoptosis and breakdown of CNS specific substrates such as dystroglycan, a transmembrane receptor involved in anchoring of astrocyte endfeet to the basement membrane via laminin binding [29]. However, substrate overlap exists as dystroglycan is also cleaved by MMP-9 with *Mmp2Mmp9 *null mice protected from neuroinflammation in an EAE model [29]. Cellular influx into areas of inflammation is regulated by MMP-2 activity. For example, HIV infected microglia/macrophages secreted proMMP-2 which is activated by neuronal MMP-14 and cleaves astrocyte-derived CXCL12 (SDF-1) that is itself neurotoxic and HIV-infected leucocytes up-regulate MMP-2 (and -9) secretion in order to cross the blood brain barrier (BBB) [10,30]. MMP-2 is thus an effector of tissue destruction in the CNS and mechanisms that lead to its suppression may be neuroprotective.

There are few published data on MMP-2 in TB but we and others have shown that cerebrospinal fluid concentrations are raised in TBM with no concomitant rise in TIMP-1 or -2 [21,31,32]. In addition, MMP-2 has been detected by immunohistochemistry in meninges of CNS TB patients [31]. In CNS TB excess MMP-2 is likely to be secreted by microglial cells, the resident innate cells of the CNS, as TB infected monocytes/macrophages do not secrete MMP-2 and astrocytes do not alter MMP-2 secretion in response to TB infection [11,12]. Therefore we investigated mechanisms regulating MMP-2 gene expression and secretion in a simplified cellular model of CNS TB.

## Methods

Chemicals for microglial inhibition, including the caspase 8 inhibitor (z-IETD-fmk) were from Sigma-Aldrich (Gillingham, UK), tissue culture materials from Invitrogen (Paisley, UK) and tissue culture plastic from TPP (Trasadingen, Switzerland) unless otherwise stated. Anti-TNF-α neutralizing antibodies, recombinant TNF-α, IL-1Ra, recombinant IL-1β were from Peprotech (Rocky Hill, NJ, US). SB203580, PD98059, SP600125 and SC-514 were purchased from Calbiochem (Merck, Nottingham, UK), helenalin from Biomol (Exeter, UK).

### M.tb culture and colony counting

The virulent *M.tb *strain H37Rv Pasteur was cultured from frozen stocks in Middlebrook 7H9 broth with 10% OADC enrichment medium (BD Biosciences, Oxford, UK), 0.2% glycerol, and 0.02% Tween 80 in a shaking incubator. An optical densitometer (Biowave Cell Density Meter, WPA, Cambridge, UK) was used to assess growth of *M.tb*. Experiments were performed with cultures having an OD of 0.6 after 30 seconds sonication. The endotoxin level was <0.03 ng/ml LPS as measured by amoebocyte lysate assay (Associates of Cape Cod, East Falmouth, MA, US). Multiplicity of infection (MOI) used in each experiment was checked by colony counts on Middlebrook 7H11 agar containing ADC enrichment medium (BD Biosciences) and amphotericin 2.5 μg/ml. Tb medium was generated by centrifuging *M.tb *at OD 0.6 at 11,700 RCF (13,000 RPM) for 5 minutes and then sterile filtering the supernatant through an Anopore 0.2 mM membrane (Anopore, Brentford, UK).

### Monocyte isolation and M.tb infection

PBMCs were isolated from single healthy blood donor residual buffy coats (UK Blood Transfusion Service) using Ficoll-Paque (GE Healthcare, Little Chalfont, UK) density gradient centrifugation and a standard adherence protocol [11]. Monocytes were stimulated by *M.tb *at an MOI of 1 or 7H9 medium alone. At 24 hours, cell culture medium was collected and filtered via a 0.2-μm pore size sterile filter (Anopore) to remove infectious particles. This conditioned medium from *M.tb *infected monocytes was termed CoMTb and conditioned medium from uninfected, control monocytes termed CoMCont. Similarly CoACont and CoATb were produced from infecting primary human astrocytes with *M.tb *but at MOI of 10 in serum free Modified Eagle's Medium (MEM) after preliminary experiments showed no effect of an MOI of 1 (data not shown) and no excess cell death with an MOI of 10.

Human CHME3 microglial cells (a gift of Professor Marc Tardieu, Paris, France and donated by Dr Nicola Woodroofe, Sheffield Hallam University, UK) were maintained in Dulbecco's Modified Eagle's Medium (DMEM) supplemented with 10% FCS (Biosera, Ringmer, UK) and 3 mM glutamine in a humidified incubator with 5% CO2 at 37°C. For experiments cells were seeded the day previously at 35-50,000 cells/cm^2 ^(to ensure that MOI was little affected by replication as previous data confirmed no change in infectivity [33]) and performed in macrophage serum free medium (MSFM). Cell culture medium was not replaced and harvested at specified time points, centrifuged for 5 minutes at 12,000 g to remove cellular debris and samples frozen at -20°C for later analysis. In direct infection experiments, *M.tb *was removed by filtration of all samples through a 0.2-μm pore size Durapore sterile filter (Millipore, Watford, UK) to remove infectious particles but retain MMP activity [34].

### Gelatin zymography

Zymography is substrate based SDS-PAGE electrophoresis which provides data on all the potentially active MMP present in the sample analyzed [35]. Enzyme activity is seen as white bands on Comassie blue stained gels [36]. A 0.2 ng MMP-9 standard (Oncogene, CA, US) was run on each gel to compensate for gel to gel variability and gels run as previously described [37]. Gels were analyzed densitometrically by digital image capture (UVP Transilluminator, Cambridge, UK) and by Scion Image Analysis software (NIH Image version 1.61, Bethesda, MD, US).

### Analysis of MMP-2 by Luminex multianalyte technology

Total immunoreactive MMP-2 concentrations (pro-, active and degraded) were analyzed for MMP-2 secretion using Fluorokine multianalyte profiling kits (R&D Systems, Abingdon, UK) and the Luminex platform Bio-Plex 200 system (Bio-Rad, Watford, UK). Bio-Plex manager software (version 5.0) was used to construct standard curves and calculate unknowns. The minimum level of detection for MMP-2 was 80 pg/ml.

### Gene expression analysis by real time quantitative PCR

Microglia were lysed with TRI Reagent and total RNA was extracted using a standard chloroform-phenol-isopropanol protocol using phase-lock-gel tubes (Eppendorf, Cambridge, UK) [38]. RNA was further purified by a DNAse step using a commercial kit (Ambio, Austin TX, US). Reverse transcription of 1 μg RNA was followed by quantitative polymerase chain reactions (qPCRs) performed in a 25 μL reaction with a Stratagene Mx3000P machine (Stratagene, La Jolla, CA, US) using 5 ng cDNA, Brilliant II qPCR mastermix (Stratagene) and MMP primers and probes as described previously [39]. The cycle threshold (C_T_) at which amplification entered the exponential phase was determined and this number was used to indicate the amount of target RNA in each sample. The MMP C_T _calculated was also normalized to ribosomal 18s C_T _run concurrently.

### Western Blotting

Standard western blotting was performed. For p38 MAP kinase analysis cells were lysed using 200 μl of SDS sample buffer (62.5 mM Tris pH 6.8, 2% SDS, 10% glycerol, 50 mM DTT and 0.01% bromophenol blue) and immediately frozen at -80°C. 40 μl aliquots were run as described [40]. Overnight incubation in 1:1000 rabbit primary antibodies to both phosphorylated and unphosphorylated variants of p38 was followed by washing and incubation with 1:2000 dilution of anti-rabbit HRP-linked secondary antibody (both Cell Signaling Technology, MA, US). Bands were visualized by chemiluminescence with the ECL plus system (GE Healthcare) according to manufacturer's instructions. Anti-caspase 8 p18 subunit rabbit polyclonal primary antibody (Santa Cruz, CA, US) was used at 1:1000 dilution with the same secondary outlined above.

### Immunohistochemistry

Ethical consent for the study of anonymized paraffin embedded sections from the histopathology archive was obtained from the Hammersmith Hospitals Research Ethics Committee in accordance with The Human Tissue Act 2004. Formalin-fixed and paraffin embedded brain biopsies from five immunocompetent patients with biopsy-proven CNS *M.tb *infection, two negative controls (the superior frontal and the superior temporal gyri of normal post-mortem brains with no pathological changes) and two positive controls (patients who had undergone temporal lobectomy epilepsy surgery) were immunostained for the microglial marker Iba1 (rabbit polyclonal antibody, Wako, Osaka, Japan, dilution 1:400) and phospho-p38 (mouse monoclonal, Sigma-Aldrich, dilution 1:200) as well as standard H&E staining. Five μm sections were deparaffinised in xylene and rehydrated in alcohol. Endogenous peroxidase activity was blocked with 0.3% hydrogen peroxide in PBS for 30 minutes and antigen retrieval of sections was performed by steaming the sections in citrate buffer (0.01 M citrate pH 6.5) for 30 minutes. Sections were then incubated with 10% normal goat serum (Vector Laboratories, Burlinghame, CA, US) for 20 minutes at room temperature. The primary antibody was applied overnight at 4°C. The following day staining was visualized using biotinylated secondary antibodies followed by avidin-biotin peroxidase complex (Vectastain Elite ABC kit, Vector Laboratories, Burlingham, CA, US). The reaction product was revealed with 2 μg/mL 3, 3'-Diaminobenzidine (DAB) and 0.0075% hydrogen peroxide in PBS. Slides were counterstained with hematoxylin and coverslipped. Methodological negative controls included omission of the primary antibody.

### Preparation of nuclear & cytoplasmic extracts

30 minute timepoint nuclear and cytoplasmic extracts were prepared using a commercial kit (Active Motif, Rixensart, Belgium) according to the manufacturer's instructions. To ensure equal total protein loading of samples between wells in subsequent Western and ELISA assays of cell lysates a Bradford assay was used to calculate total protein concentration in samples.

### Detection of nuclear NFκB DNA binding

To investigate activation of NFκB subunits a specific transcription factor assay (TransAM, Active Motif) with primary antibodies to p50 and p65 was used. Competition experiments demonstrated specificity of binding by adding 20 pM/well of either wildtype or mutated NFκB oligonucleotide before assaying with the p65 antibody and demonstrating loss of binding with wildtype but not mutated construct. Values are expressed as fold change normalized to control conditions.

## Results

### Microglial MMP-2 secretion is suppressed more by CoMTb than by direct infection

**Figure 1:**
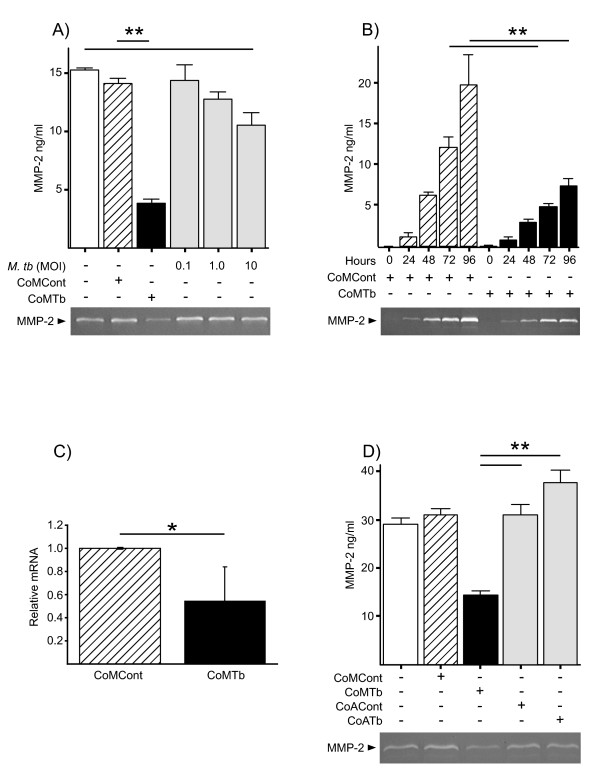
**MMP-2 secretion and gene expression is suppressed by CoMTb**. (**A**), Microglial cells were stimulated with control medium (open bars), CoMCont (diagonal hatched bars), CoMTb (solid bars) or infected with *M.tb *(grey bars) at an MOI of 1-10. 72 h supernatants were analyzed by Luminex with a representative gelatin zymogram shown. (**B**), Kinetics of CoM stimulated MMP-2 secretion analyzed by Luminex (representative zymogram shown). (**C**), MMP-2 gene expression is suppressed by CoMTb. mRNA from microglia stimulated for 24 h was analyzed by RT-PCR, normalized to 18S RNA and expressed as fold change relative to 24 h CoMCont mRNA. The mean ± SD from 3 experiments are shown analyzed by Student's t-test. (**D**), Astrocyte-microglial signaling networks do not suppress MMP-2 secretion. Conditioned medium from primary human astrocytes was prepared from control (CoACont) and *M.tb *infected cells (CoATb) and used to stimulate microglia. 72 h cell culture medium MMP-2 secretion was analyzed by Luminex (representative zymogram shown). A, B & D, Bars represent mean values ± SD of 3 samples, representative of at least duplicate experiments performed in triplicate. Data were analyzed by one-way analysis of variance, followed by Tukey's multiple comparison. *p < 0.05, **p < 0.01.

Microglia were stimulated with 1:5 diluted CoMCont, CoMTb or infected with *M.tb *at MOIs of 0.1, 1 and 10. CoMTb caused a 72.8% suppression of MMP-2 secretion (Figure 1A, P < 0.01). There was a 31% decrease in MMP-2 secretion due to direct infection of microglia at an MOI of 10 compared to control (P < 0.01), an infectious load-dependent effect but no effect on cell viability was demonstrated. CoMTb suppression of MMP-2 was evident by 24 hours, significant by 72 hours and sustained over 96 hours (Figure 1B). CoMTb decreased MMP-2 mRNA accumulation by 50% at 24 hours (Figure 1C, P < 0.05). This effect of CoMTb on microglia was specific since conditioned media from *M.tb *infected primary human astrocytes (CoATb, Figure 1D) had little effect and microglia (data not shown) had no effect on MMP-2 secretion.

### TNF-α but not IL-1β in CoMTb suppresses microglial MMP-2 secretion

**Figure 2:**
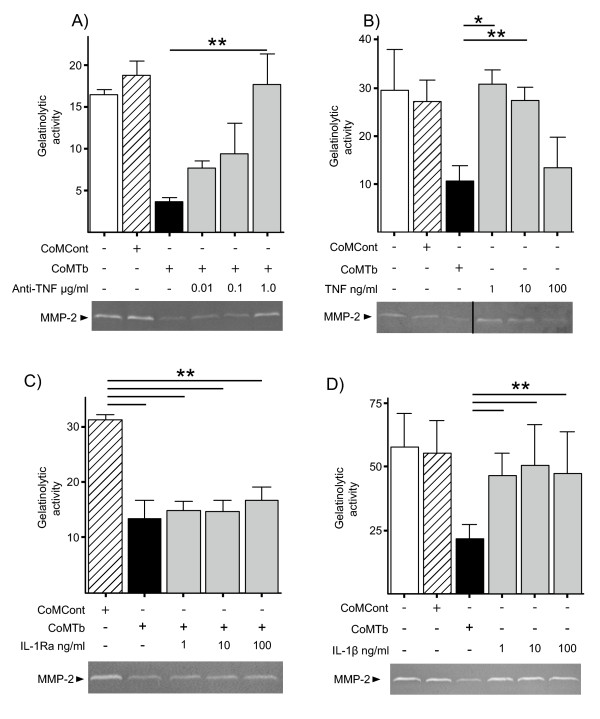
**Suppression of MMP-2 by CoMTb is mediated by TNF-α**. (**A**), CoMTb was preincubated with increasing doses of anti-TNF-α Ab for 2 h. (**B**), TNF-α suppresses MMP-2 secretion. Cells were stimulated with rhTNF-α 1-100 ng/ml. Note representative gelatin zymogram has been cut to correspond to the adjacent graph (thick black line). (**C**), IL-1β does not contribute to CoMTb induced MMP-2 secretion. Cells were preincubated with IL-1Ra for 2 h. Inhibition of IL-1β activity did not affect MMP-2 secretion. (**D**), IL-1β alone does not suppress MMP-2 secretion. Microglial cells were stimulated with rhIL-1β at concentrations of 1-100 ng/ml. Densitometric analysis of gelatin zymography is shown with a representative gelatin zymogram for A-D. For all experiments bars represent mean values ± SD of three samples, representative of at least duplicate experiments performed in triplicate. Data were analyzed by one-way analysis of variance followed by Tukey's multiple comparison. *p < 0.05, **p < 0.01.

The effect of pre-incubation of CoMTb for two hours with increasing concentrations of TNF-α neutralizing antibody before stimulating microglia was investigated (Figure 2A). Previously we have demonstrated that a matched isotype control antibody has no inhibitory activity [41]. MMP-2 secretion was restored in a dose-dependent manner. 100 ng/ml recombinant TNF-α suppressed MMP-2 secretion by 61.0% similar to the level observed with CoMTb (Figure 2B). As we have shown that the TNF-α concentration in 1:5 CoMTb is approximately 2 ng/ml [33,37] the data show that TNF-α is necessary, but not sufficient, to cause CoMTb-induced MMP-2 suppression. Next, we investigated IL-1β which is present in CoMTb and important in CNS TB pathogenesis [20]. Pre-incubating microglial cells with the inhibitor IL-1Ra or stimulation with recombinant IL-1β did not alter MMP-2 secretion (Figure 2C &2D). There was neither an additive nor synergistic effect of adding these two recombinant cytokines concurrently (Additional file 1A). Soluble factors derived from *M.tb *culture (Tb medium) did not synergize with TNF-α to further suppress MMP-2 secretion since we demonstrated that there was no additional effect of adding filtered supernatant from cultured *M. tuberculosis *to TNF-α (Additional file 1B). Dexamethasone also did not regulate MMP-2 secretion (Additional file 2) nor did addition of IL-6, Oncostatin M or inhibition of G-protein coupled signaling via pertussis blockade experiments (data not shown).

### p38 and ERK MAPK divergently regulate CoMTb-mediated MMP-2 secretion

**Figure 3:**
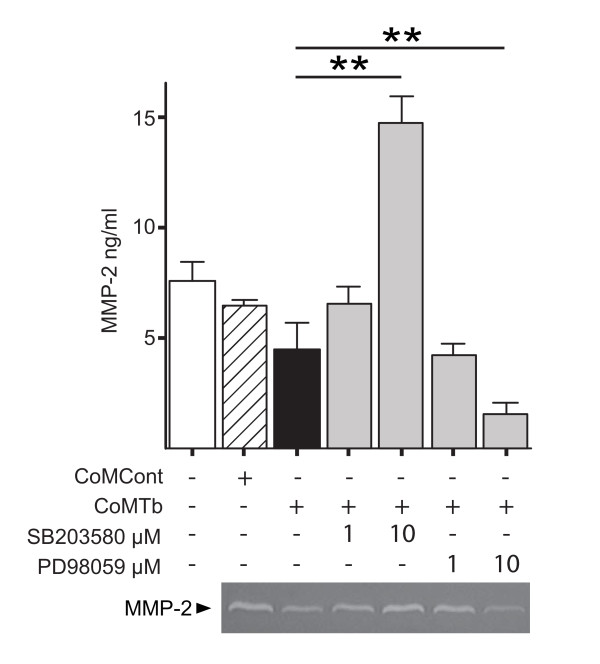
**p38 phosphorylation acts as the break for CoMTb suppression of MMP-2 secretion**. Microglial cells were incubated with SB203580 or PD98059 for 2 h, and then stimulated with CoMTb. MMP-2 secretion was analyzed at 72 h by Luminex and gelatin zymography (representative gel shown). p38 inhibition reverses CoMTb suppression and leads to supra-maximal MMP-2 secretion but ERK inhibition further suppresses secretion. Bars represent mean values ± SD of three samples, representative of at least duplicate experiments performed in triplicate. Data were analyzed by one-way analysis of variance, followed by Tukey's multiple comparison. **p < 0.01.

Inhibition of the p38 pathway abrogated CoMTb-mediated MMP-2 suppression (Figure 3). 1 μM SB230580 caused CoMTb-driven MMP-2 secretion to return to the same level as constitutively secreted and 10 μM SB230580 caused a 228% increase in MMP-2 secretion over CoMCont (P < 0.01). In contrast ERK inhibition at 1 μM PD98059 caused a 6.1% decrease in MMP-2 secretion over and above the 31.2% reduction due to CoMTb (P < 0.05). 10 μM PD98059 caused a significant 76.6% decrease in MMP-2 secretion compared to CoMCont (P < 0.01). JNK inhibition with 1 μM SP600125 led to a small but non-significant further decrease in MMP-2 secretion but 10 μM caused cell death (data not shown).

**Figure 4:**
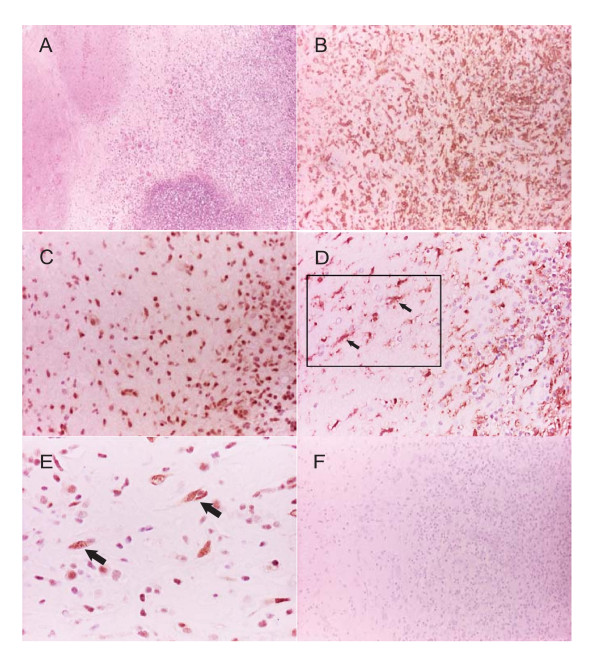
**Microglia are likely to express phosphorylated p38 in patients with CNS TB**. Brain biopsies from five patients with CNS TB were stained. (**A**) H&E stained section of cerebellar cortex shows typical TB necrotizing granulomas (original magnification 10×). (**B**) Iba1 (a microglial/macrophage marker) staining confirms a florid microglial and macrophage infiltrate in the TB granuloma (original magnification 20×). (**C**) p38 immunoreactivity is almost exclusively expressed in the nucleus of microglial cells (original magnification 40×). (**D**) Iba-1 staining in microglia denoted by arrows with rectangle denoting area enlarged for (E) (original magnification 20×) (**E**) corresponding p38 positive microglia with characteristic nuclear shape enlarged from rectangle in (D) (original magnification 40×). For immunostaining (B - E) areas of immunoreactivity appear as brown against the blue counterstain. (**F**) Omission of the primary antibody in CNS TB biopsy demonstrates no immunoreactivity (original magnification 20×).

Since p38 appeared to have a potentially key role in CoMTb-induced MMP-2 suppression, the expression of microglial phospho-p38 in five CNS TB brain biopsies was investigated for the first time in human brain sections. Microglial cells appeared to be immunoreactive for phosphorylated p38 (Figure 4). Specifically nuclei were more immunoreactive for phosphorylated p38 than cytoplasm. Negative control brains and relevant methodological negative controls, including omission of primary antibody, did not demonstrate any p38 immunoreactivity further strengthening these novel data.

### NFκB signalling suppresses MMP-2 gene expression

**Figure 5:**
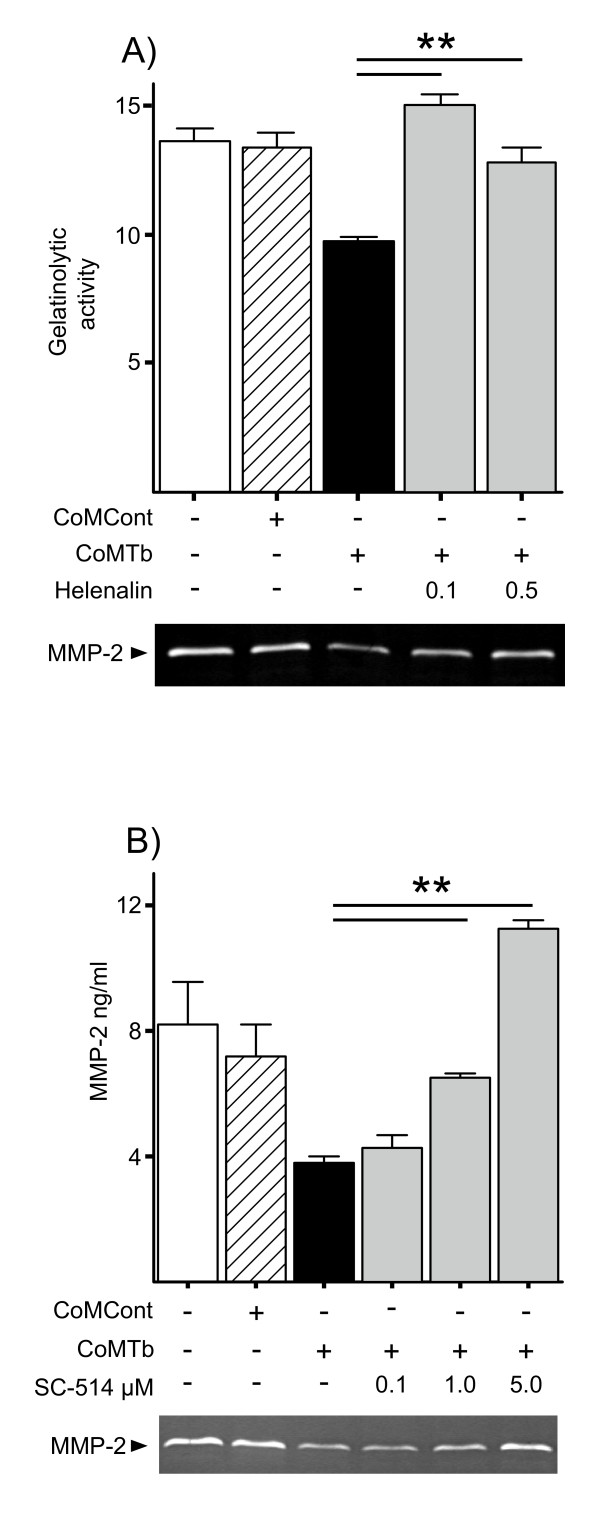
**CoMTb suppression of MMP-2 secretion is mediated by the NFκB pathway**. (**A**), Cells were preincubated with helenalin for 2 h and then stimulated with CoMTb. 72 h cell culture supernatants were analyzed by Luminex, confirmed by gelatin zymography (representative zymogram shown). (**B**), SC-514 inhibition. Bars represent mean values ± SD of three samples, representative of at least duplicate experiments performed in triplicate. Data were analyzed by one-way analysis of variance, followed by Tukey's multiple comparison. **p < 0.01.

Since NFκB is a key regulator of many MMPs, the effect of p65 blockade was investigated. Helenalin restored MMP-2 secretion to basal levels (Figure 5A). Confirmation of these data was obtained by inhibition of IKK2 with SC-514 which resulted in similar dose-dependent loss of CoMTb mediated MMP-2 suppression (Figure 5B). 5 μM SC-514 resulted in MMP-2 concentrations that were 157% higher than CoMCont and 301% higher than CoMTb (both P < 0.01). No such increased secretion was observed with helenalin since this compound was toxic to cells at concentrations greater than 1 μM.

### Caspase 8 inhibition restores CoMTb-induced MMP-2 secretion to control concentrations

**Figure 6:**
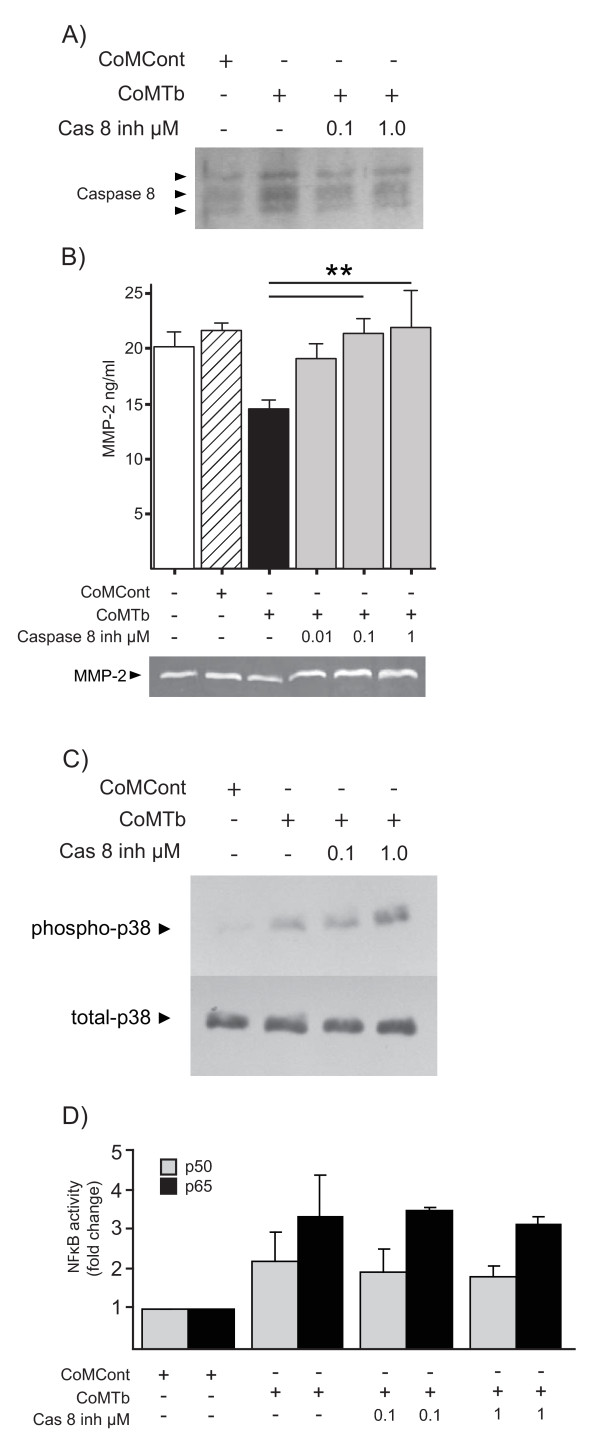
**Caspase 8 mediates CoMTb induced MMP-2 suppression**. (**A**) Caspase 8 activity is up-regulated by CoMTb at 30 minutes and returned to baseline by the inhibitor z-IETD-fmk. Representative Western blot shown. (**B**) Caspase 8 inhibition restores CoMTb-mediated MMP-2 suppression. 72 h supernatants were analyzed by Luminex and confirmed by gelatin zymography. Bars represent mean values ± SD of three samples, representative of at least duplicate experiments performed in triplicate. Data were analyzed by one-way analysis of variance, followed by Tukey's multiple comparison. **p < 0.01. (**C**) Phospho-p38 activity at 30 minutes is up-regulated by caspase 8 inhibition. Representative Western blot shown. Total p38 blot to show equal lane loading. (**D**) Caspase 8 inhibition does not alter NFκB nuclear dynamics. Nuclear extracts of CoM stimulated cells were taken at 30 mins and p50 & p65 subunits measured by ELISA.

We next studied caspase-8 which has been proposed to have a non-apoptotic role in regulating MMP secretion [25]. Caspase 8 activity was increased at 30 minutes by CoMTb and was returned to baseline by the caspase 8 inhibitor z-IETD-fmk (Figure 6A). Cells were then pre-incubated with the caspase 8 inhibitor and stimulated with CoMTb after two hours. A dose-dependent increase in MMP-2 secretion was observed with increasing concentrations of caspase 8 inhibitor, with MMP-2 secretion returned to basal levels after pre-treatment of cultures with 1 μM z-IETD-fmk (Figure 6B, P < 0.01).

Next, we investigated the influence of the caspase 8 pathway on p38 MAPK. Caspase 8 inhibition led to a paradoxical increase in p38 phosphorylation at 30 minutes (Figure 6C). There were no differences in cell numbers during these experiments. Finally, we investigated whether caspase 8 might act via the NFκB pathway. Both p50 and p65 NFκB subunits were up-regulated in nuclear extracts from CoMTb-stimulated microglial cells by 30 minutes. However, caspase 8 acts independently of p65/p50 NF-kB nuclear translocation (Figure 6D).

## Discussion

In this study, we demonstrated that a TNF-dependent cytokine network involving *M.tb*-infected monocytes, but not direct infection by *M.tb*, down-regulates MMP-2 secretion from microglial cells via p38 MAP kinase, NFκB and caspase 8 pathways. Since *M.tb *infection and TNF-α usually drive gene expression and secretion of other MMPs, such as MMP-1, -3 and -9, leading to pro-inflammatory damage this was an unexpected, paradoxical finding [33,37,42]. Specifically this was the only down-regulated molecule when studying all MMPs in this model system in our previously published work and in particular contrast to the other gelatinase MMP-9 [33]. p38 staining has never previously been demonstrated *in vivo *in human microglia. Caspase 8 has not been implicated in the regulation of MMP secretion in TB.

In our simplified cellular model of CNS TB, MMP-2 secretion was suppressed up to 96 hours by CoMTb more profoundly than by *M.tb *alone. The effect of monocyte-microglial networks was specific since neither astrocyte nor microglial-derived conditioned medium affected MMP-2 secretion. These data, albeit from a cell line, are consistent with observations that MMP-2 was not detected in parenchymal cells in CNS TB suggesting that cell specific down-regulation may be occurring *in vivo *[31,32]. Control of MMP-2 activity is usually achieved by restricting the activation of the secreted pro-enzyme [18]. However, in microglial cells decreased MMP-2 secretion was mainly due to inhibition of MMP-2 mRNA accumulation, a phenomenon only previously reported in astrocytoma cells [43]. It is possible that this finding is specific to the CNS immune response.

TNF-α was necessary, but not sufficient for MMP-2 suppression as 50 times the concentration of recombinant molecule was needed to give the equivalent CoMTb effect. We and others have previously demonstrated that TNF-α is often a critical up-regulator of MMP expression and secretion both in TB and other model systems [13,25,40]. However, TNF-α has been reported to down-regulate expression and secretion of MMP-2 from both astrocytoma and choroid plexus epithelial cells [43,44]. In contrast IL-1β had no effect on MMP-2 secretion although in our previous work we demonstrated that this cytokine drives astrocyte MMP-9 secretion [45]. Dexamethasone tends to down-regulate proinflammatory responses and has a clinically important adjuvant role in decreasing inflammation in the treatment of CNS TB [46]. Dexamethasone did not reverse the paradoxical effect of CoMTb on MMP-2 gene expression. Similarly in a rat meningitis model dexamethasone did not influence parenchymal MMP-2 expression suggesting the MMP-2 gene may not be steroid responsive in the CNS [47].

We investigated the role of ERK and p38 MAP kinase pathways in MMP-2 down-regulation. ERK inhibition further inhibited MMP-2 secretion. In contrast p38 MAP kinase acts as a brake to MMP-2 secretion as pre-incubation of cells with the specific p38 inhibitor SB203580 leading to supra-maximal secretion of MMP-2. The p38 MAP kinase pathway is a critical regulator of inflammatory processes [22]. Our group has demonstrated that p38 acts as a brake in a similar way in pulmonary epithelial cells [40]. To strengthen these *in vitro *findings, we investigated the expression of phospho-p38 in biopsies from proven human CNS TB patients and showed likely parenchymal activation of this pathway in microglia *in vivo *for the first time, an important finding from this work. It is not possible using any currently available markers to be certain whether these positive cells were resident microglia or infiltrating peripheral macrophages [48].

Blockade of the NFκB pathway with SC-514 restored CoMTb inhibited MMP-2 secretion to supra-maximal levels. Similarly, helenalin opposed the effect of CoMTb on MMP-2. Down-regulation of gene expression by the NFκB pathway has been previously described in other secretory pathways, such as apolipoprotein E mRNA in adipocytes via p50 homodimer binding to the promoter, but this is the first time that control of MMP-2 secretion has been shown to be modulated in this way in the CNS [49,50]. In contrast many other MMP genes are known to have NFκB responsive elements in their promoters that act as up-regulators of gene transcription [51,52].

Importantly we showed, for the first time, that caspase 8 inhibition restored CoMTb-induced MMP-2 secretion in human microglial cells in a dose dependent fashion independent of the p38 and NFκB pathways. Although p38 signaling was reported as essential for caspase-induced apoptosis in macrophages, we were unable to demonstrate p38 driven caspase 8 activation in our system and blockade did not alter cell numbers [53]. Non-apoptotic roles for caspase 8 including NFκB activation have been described but here caspase 8 blockade had no effect on nuclear recruitment of p50 and p65 NFκB subunits [54]. In contrast caspase 8 may mediate TNF-α activation of NFκB via a p38 independent pathway involving FLICE-associated huge protein (FLASH)-caspase 8 complexes in HeLa and HEK293 cells [55]. Caspase-2 cleavage is also downstream of TNF-α receptor activation and has demonstrated non-apoptotic roles including cell matrix adhesion, as do caspase 3 and 7 [56,57]. Finally, although caspase 8 activity was demonstrated to be rapidly induced by CoMTb, the effect on MMP-2 secretion was more delayed which suggest that it is probable that there are additional intermediary events occurring in this pathway which are not yet characterized.

In summary, there is a decrease in microglial MMP-2 secretion in response to networks driven by *M.tb*-infected monocytes, which coupled with the known rise in the acknowledged effector molecule MMP-9 is likely to lead to a change in the CNS inflammatory milieu characterized by alterations in chemokine and cytokine processing as well as cellular migration [58]. Unexpectedly, such networks are paradoxically dependent upon the pro-inflammatory cytokine TNF-α (but not IL-1β). Within microglia, the p38 MAP kinase, NFκB p65 subunits and caspase 8 pathways are all involved in the control of MMP-2 down-regulation. Caspase 8, which to date has been more implicated in regulation of apoptosis in infected cells, acts independently from a separate p38 MAP kinase/NFκB axis to suppress MMP-2 secretion in microglial cells in this simplified cellular model of CNS TB.

## Competing interests

The authors declare that they have no competing interests.

## Authors' contributions

JAG, SD, RM, CWMO and JF designed, performed and analyzed experiments. JAG, JSF and PTE interpreted the results. KJ and FR performed and interpreted the immunohistochemistry. All of the authors contributed to the writing of the manuscript. All authors have read and approved the final version of the manuscript.

## Supplementary Material

Additional file 1 **Figure S1**. (**A**) CoMTb suppression of MMP-2 secretion is not mediated by IL-1β and TNF-α synergy. Microglia were stimulated with TNF-α and IL-1β either alone or in combination. There was no additional effect of adding IL-1β to TNF-α. (**B**) CoMTb suppression of MMP-2 secretion is not mediated by *M.tb *antigens and TNF-α synergy. Microglia were stimulated with TNF-α 100 ng/ml, Tb medium (Tb med 4.7 μl/ml), either alone or in combination. No effect was seen on MMP-2 secretion. 72 h supernatants were analyzed by Luminex. Bars represent mean values ± SD of three samples, representative of at least duplicate experiments performed in triplicate. Data were analyzed by one-way analysis of variance, followed by Tukey's multiple comparison. **p < 0.01. Click here for file

Additional file 2 **Figure S2**. Dexamethasone does not reverse CoMTb induced suppression of MMP-2 secretion. Cells were preincubated with dexamethasone for 2 hours then stimulated with CoMTb. 72 h supernatants were analyzed by Luminex and confirmed by gelatin zymography. Bars represent mean values ± SD of three samples, representative of at least duplicate experiments performed in triplicate. Data were analyzed by one-way analysis of variance, followed by Tukey's multiple comparison. **p < 0.01. Click here for file
