# Supplementary material for: Mycobacterium tuberculosis-infected human monocytes down-regulate microglial MMP-2 secretion in CNS tuberculosis via TNFα, NFκB, p38 and caspase 8 dependent pathways
Source: J Neuroinflammation. 2011 May 11;8:46. doi: 10.1186/1742-2094-8-46 (PMC3113956; doi:10.1186/1742-2094-8-46)
Supplement: Additional file 1 — Figure S1. (A) CoMTb suppression of MMP-2 secretion is not mediated by IL-1β and TNF-α synergy. Microglia were stimulated with TNF-α and IL-1β either alone or in combination. There was no additional effect of adding IL-1β to TNF-α. (B) CoMTb suppression of MMP-2 secretion is not mediated by M.tb antigens and TNF-α synergy. Microglia were stimulated with TNF-α 100 ng/ml, Tb medium (Tb med 4.7 μl/ml), either alone or in combination. No effect was seen on MMP-2 secretion. 72 h supernatants were analyzed by Luminex. Bars represent mean values ± SD of three samples, representative of at least duplicate experiments performed in triplicate. Data were analyzed by one-way analysis of variance, followed by Tukey's multiple comparison. **p < 0.01. [file 1742-2094-8-46-S1.PDF]

Additional file 1

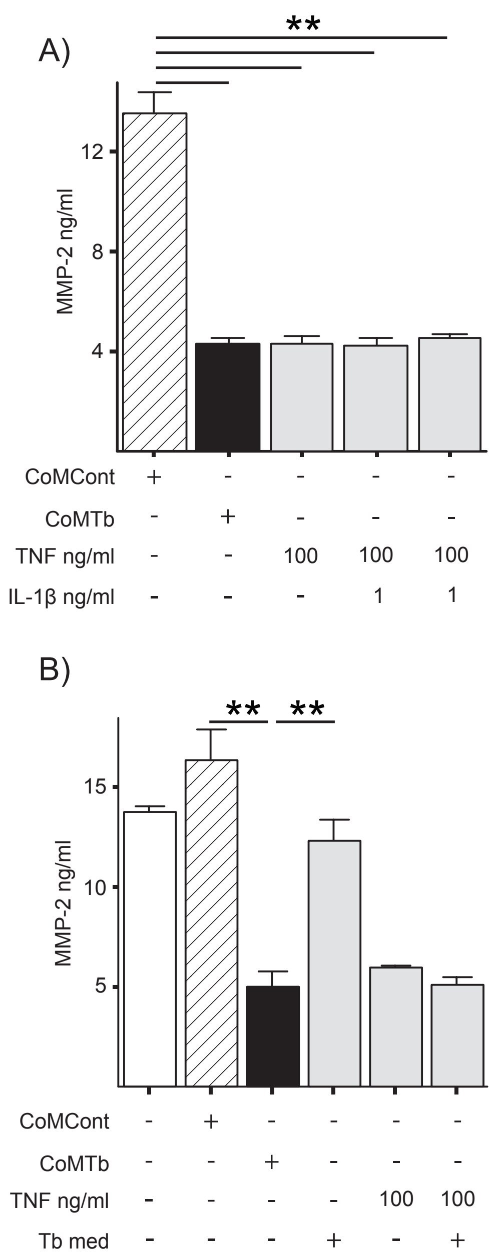

(A) CoMTb suppression of MMP-2 secretion is not mediated by IL-1β and TNF-α synergy. Microglia were stimulated with TNF-α and IL-1β either alone or in combination. There was no additional effect of adding IL-1β to TNF-α. (B) CoMTb suppression of MMP-2 secretion is not mediated by M.tb antigens and TNF-α synergy. Microglia were stimulated with TNF-α 100 ng/ml, Tb medium (Tb med 4.7 μl/ml), either alone or in combination. No effect was seen on MMP-2 secretion. 72 h supernatants were analyzed by Luminex. Bars represent mean values ± SD of three samples, representative of at least duplicate experiments performed in triplicate. Data were analyzed by one-way analysis of variance, followed by Tukey's multiple comparison. **p<0.01.
