# Supplementary material for: Mycobacterium tuberculosis-infected human monocytes down-regulate microglial MMP-2 secretion in CNS tuberculosis via TNFα, NFκB, p38 and caspase 8 dependent pathways
Source: J Neuroinflammation. 2011 May 11;8:46. doi: 10.1186/1742-2094-8-46 (PMC3113956; doi:10.1186/1742-2094-8-46)
Supplement: Additional file 2 — Figure S2. Dexamethasone does not reverse CoMTb induced suppression of MMP-2 secretion. Cells were preincubated with dexamethasone for 2 hours then stimulated with CoMTb. 72 h supernatants were analyzed by Luminex and confirmed by gelatin zymography. Bars represent mean values ± SD of three samples, representative of at least duplicate experiments performed in triplicate. Data were analyzed by one-way analysis of variance, followed by Tukey's multiple comparison. **p < 0.01. [file 1742-2094-8-46-S2.PDF]

Additional file 2

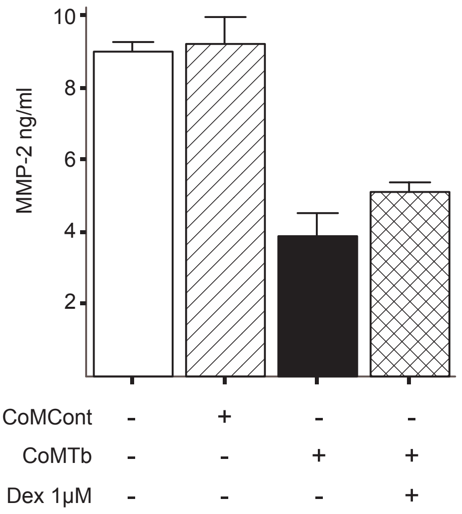


Dexamethasone does not reverse CoMTb induced suppression of MMP-2 secretion. Cells were preincubated with dexamethasone for 2 hours then stimulated with CoMTb. 72 h supernatants were analyzed by Luminex and confirmed by gelatin zymography. Bars represent mean values ± SD of three samples, representative of at least duplicate experiments performed in triplicate. Data were analyzed by one-way analysis of variance, followed by Tukey's multiple comparison. **p<0.01.
